# N6-Methyladenosine Writer Gene ZC3H13 Predicts Immune Phenotype and Therapeutic Opportunities in Kidney Renal Clear Cell Carcinoma

**DOI:** 10.3389/fonc.2021.718644

**Published:** 2021-08-23

**Authors:** Tao Guo, Hongxiang Duan, Jinbo Chen, Jinhui Liu, Belaydi Othmane, Jiao Hu, Huihuang Li, Xiongbing Zu

**Affiliations:** ^1^Department of Urology, Xiangya Hospital, Central South University, Changsha, China; ^2^School of Computer Science and Engineering, Central South University, Changsha, China

**Keywords:** ZC3H13, RNA modification N6-methyladenosine, kidney renal clear cell carcinoma, tumor immune microenvironment, immunotherapy

## Abstract

**Background:**

Although the RNA modification N6-methyladenosine ZC3H13 has been found to play vital regulatory roles in many types of cancers, its role in predicting the tumor immune microenvironment (TME) and response to immune checkpoint blockade (ICB) in kidney renal clear cell carcinoma (KIRC) remains unclear.

**Methods:**

We comprehensively analyzed the expression, prognostic significance and immunological role of ZC3H13 in pan-cancers and systematically correlated ZC3H13 with TME cell-infiltration, ICB response and targeted therapy in KIRC. The data were collected from The Cancer Genome Atlas (TCGA), Gene Expression Omnibus (GEO), Genotype-Tissue Expression (GTEx), Broad Institute Cancer Cell Line Encyclopedia (CCLE) and DrugBank database. Also, we performed RNA sequencing (RNA-seq) of 46 renal cell carcinoma tissues and 11 adjacent normal tissues to validate our result. All analyses were implemented using R software, version 3.6.3.

**Results:**

ZC3H13 was significantly differentially expressed in most tumors. However, its expression profiles and prognostic significance were consistent only in KIRC, regardless of overall survival, progression-free survival and cancer-specific survival. Additionally, ZC3H13 expression was correlated with clinicopathological factors in KIRC. Furthermore, we found that ZC3H13 might shape a noninflamed phenotype and could predict a lower response to ICB in KIRC. These results could be validated in our own RNA-seq data. Tumor mutation burden (TMB) was significantly higher in the low ZC3H13 group. Finally, we found that ZC3H13 could predict the sensitivity of targeted therapy for KIRC.

**Conclusions:**

ZC3H13 might shape a noninflamed phenotype in KIRC. Moreover, ZC3H13 could predict the prognosis and clinical response of ICB and the sensitivity to targeted therapies in KIRC.

## Background

Kidney renal clear cell carcinoma (KIRC) is one of the most common cancers of the urinary system (1). The prognosis of early-stage KIRC is favorable, while advanced KIRC is associated with an extremely poor prognosis. Targeted therapy is the most important treatment option for advanced KIRC, but improvements in its efficacy has encountered bottlenecks in recent years (1). With the development of anticancer immune checkpoint blockade (ICB), an increasing number of clinical trials have suggested that KIRC is sensitive to ICB (2–4). ICB can significantly improve the overall survival of patients who are resistant to targeted therapy. Therefore, ICB has also become an important treatment option for advanced KIRC. However, similar to other treatment options, only a portion of patients are sensitive to ICB treatment (5). It is vital to find reliable predictors of ICB efficacy considering the economic burden and fatal side effects.

RNA modification of N6-methyladenosine (m6A) is the most prominent and abundant RNA modification pattern in eukaryotic cells (6). An increasing number of studies have shown that m6A has an important regulatory role in tumor immune regulation and ICB resistance (7). ZC3H13 (zinc finger CCCH domain-containing protein 13) is an m6A writer gene. ZC3H13 is a potential regulator of nuclear RNA m6A methylation and mouse embryonic stem cell self-renewal (8). The role of ZC3H13 in carcinomas is still not clear. It has been reported that ZC3H13 could serve as a tumor suppressor gene that inhibits the proliferation of colon cancer cells by inhibiting the RAS-ERK pathway (9). However, some studies have shown that ZC3H13 could act as an oncogene to activate the NF-kB signaling pathway to promote tumor proliferation and invasion (10, 11). Currently, there are no studies elaborating the role of ZC3H13 in KIRC, especially its relationship with tumor immune characteristics.

In this study, we first explored the expression pattern and prognostic value of ZC3H13 in pan-cancers and its relationship with immune characteristics through pan-cancer analysis. Next, we performed synthetic analysis and then focused on KIRC. Finally, we further explored the predictive value of ZC3H13 for immune phenotypes and therapeutic sensitivities in KIRC.

## Methods

### Data Retrieval and Preprocessing

The R package “TCGAbiolinks” was used to download the RNA sequencing data (FPKM values) and clinical data of TCGA-KIRC from the Genomic Data Commons (GDC, https://portal.gdc.cancer.gov/) (12). Then, we transformed the FPKM values into transcripts per kilobase million (TPM) values. The pan-cancer RNA sequencing data (FPKM values), somatic mutation data, and survival information were downloaded from the UCSC Xena data portal (https://xenabrowser.net) (13). The TMB data was calculated by using VarScan2. The microsatellite instability (MSI) data were collected from the supplementary files of Bonneville’s study (14). In addition, we also downloaded the RNA sequencing data of normal tissues in the GTEx (https://www.gtexportal.org/home/) database and the RNA sequencing data of cancer cells in the CCLE (https://portals.broadinstitute.org/ccle) database. To compare the drug sensitivities between different ZC3H13-expression groups, we collected common anticancer drugs and their target genes from the DrugBank database (www.drugbank.ca). The expression matrix of GSE53757 (15) was downloaded using the “GEOquery” package and then transformed gene symbols using GPL570. Single-cell RNA-seq (scRNA-seq) data of six adjacent normal tissues was downloaded from the supplementary file of GSE159115 (16). Main clinical information of the included cohorts was summarized in **Supplementary Table 1**. Also, we summarized the clinicopathological characteristics of TCGA-KIRC patients according to the expression of ZC3H13 in **Supplementary Table 2**.

### Analysis Procedures of scRNA-seq

Following the guide reported by Luecken et al. (17), we used the “Seurat” v4.0.1 package to analyze and visualize scRNA-seq data. For quality control, we filtered out the data with unique molecular identifiers (UMIs) fewer than 500, or fewer than 250 genes, or mitochondrial ratio more than 0.20. Then, we normalized and checked the cell cycle phase based on the filtered data. We chose the top 2000 variable genes to create anchors using the “FindIntegrationAnchors” function and integrated the six data into a new matrix using the “IntegrateData” function. After integration, we run principal component analysis (PCA) and chose the top 40 PCs to run UMAP. Finally, we visualized the clusters with the resolution set as 0.8 and annotated the clusters using HumanPrimaryCellAtlasData() based on the “SingleR” package.

### Functional Analysis of the High and Low ZC3H13 Groups

First, the empirical Bayesian algorithm in the R package “limma” was used to identify the differentially expressed genes (DEGs) between the high and low ZC3H13 groups. Adjusted P value < 0.05 and |logFC| > 1 were set as the significance criteria for significant DEGs. Then, Gene Ontology (GO) and Kyoto Encyclopedia of Genes and Genomes (KEGG) analyses were performed by using the “ClusterProfiler” R package based on the DEGs. In addition, we collected 50 hallmark pathways that could represent most of the biological functional pathways from the MSigDB database (18). Finally, we calculated the enrichment scores of these pathways in each sample by using the ssGSEA algorithm.

### Depicting the Tumor Immune Microenvironment of KIRC

The tumor microenvironment includes tumor cells, tumor-infiltrating immune cells (TIICs), stromal cells and a series of tumor-related regulatory factors. Here, we conducted a comprehensive analysis of immune-related factors in the tumor microenvironment. First, we described the seven main steps of the antitumor immune response in the KIRC tumor microenvironment, including the release and presentation of cancer cell antigens (Steps 1 and 2), priming and activation of the immune system (Step 3), trafficking and infiltration of immune cells into tumors (Steps 4 and 5), and recognition and killing of cancer cells by T cells (Steps 6 and 7) (19). These seven steps were called cancer-immunity cycle. The vitality of these steps, which determines the direction of the antitumor immune response process in the tumor microenvironment and affects the level of infiltration of TIICs, was downloaded from the TIP (Tracking Tumor Immunophenotype) (http://biocc.hrbmu.edu.cn/TIP/) (20). The TIP is a meta-server using the ssGSEA and CIBERSORT algorithm based on specific marker gene sets (**Supplementary Table 3**), which can analyze the level of anti-cancer immunity (20). Furthermore, we calculated the infiltration level of these 22 immune cells using the ssGSEA algorithm based on the specific marker gene sets (**Supplementary Table 4**) (21).

### Calculating the Enrichment Scores of Immunotherapy Response Signatures and Stroma Signatures

Mariathsan et al. identified 19 ICB response-related gene signatures, including 18 positive signatures (such as DNA replication, Fanconi_anemia_pathway, Homologous_recombination, MicroRNAs_in_cancer, Mismatch_repair, Nucleotide_excision_repair, Oocyte_meiosis, p53_signaling_pathway, Progesterone_mediated_oocyte_maturation) and 1 negative signature (Cytokine_cytokine_receptor_interaction) (**Supplementary Table 5**) (22). In addition, we identified four stromal pathways with immunosuppressive effects from previous literature, including epithelial-mesenchymal transition (EMT) markers and the pan-fibroblast TGF-b response signature (Pan-FTBRS) (22). The ssGSEA algorithm was used to calculate the enrichment score of these signatures in individuals.

### RNA Sequencing of Renal Cell Carcinoma Samples

Forty-seven renal cell carcinoma tissues and thirteen adjacent normal tissues stored in liquid nitrogen were collected from our hospital. We called it Xiangya cohort. All the clinicopathological data of the patients were included and summarized in **Supplementary Table 6**. Total RNA was extracted from the samples using Trizol (Invitrogen, Carlsbad, CA, USA) according to the manufacturer’s instructions. Then, the quality of RNA was evaluated using NanoDrop and Agilent 2100 bioanalyzer (Thermo Fisher Scientific, MA, USA). We next constructed the mRNA library. The RNA was purified using Oligo(dT)-attached magnetic beads and then fragmented into small pieces. Random hexamer-primed reverse transcription was used to generate the first and second-strand cDNA. After adding A-Tailing Mix and RNA Index Adapters by incubating to end repair, the obtained cDNA was amplified by PCR and purified by Ampure XP Beads. The double-stranded PCR products were heated, denatured and circularized by the splint oligo sequence to get the final library. There were 46 qualified renal cell carcinoma tissues among the 47 samples and 11 qualified adjacent normal tissues among the 13 samples. Finally, the qualified samples were sequenced on a BGISEQ-500 platform (BGI-Shenzhen, China). The gene expression levels were calculated using RSEM (v1.2.12).

### Statistical Analysis

For the continuous variables, Pearson or Spearman coefficients were used to explore pairwise correlations. The median ZC3H13 expression (30.25) was applied as a cutoff value. Then, the cohort was classified into high and low ZC3H13 groups. The t-test was applied to analyze the difference between groups for variables with a normal distribution. Otherwise, the Mann-Whitney U test was applied. The Kaplan-Meier method was used to plot the survival curves for prognostic analyses, and the log-rank test was applied to estimate the statistical significance. P < 0.05 indicated a significant difference. All statistical tests were two-sided. Finally, all statistical data analyses were implemented using R software, version 3.6.3 (http://www.r-project.org).

## Results

### Expression Profiles of ZC3H13 in Pan-Cancers

We found that ZC3H13 was significantly differentially expressed in most tumors by comprehensively analyzing the expression data from the TCGA and GTEx databases (**Supplementary Figures 1A, B**). This indicated that ZC3H13 may be closely related to the occurrence and development of tumors. However, it is worth noting that ZC3H13 had significantly different expression in different tumors, and its expression might depend on the different types of tumors and the heterogeneity of the tumors. For example, the expression of ZC3H13 was significantly lower in tumor tissues than in adjacent normal tissues in KIRC, bladder urothelial carcinoma (BLCA), breast invasive carcinoma (BRCA), cervical squamous cell carcinoma and endocervical adenocarcinoma (CESC) etc. In contrast, the expression of ZC3H13 was significantly higher in the tumor tissues in cholangiocarcinoma (CHOL), esophageal carcinoma (ESCA), skin cutaneous melanoma (SKCM) etc. For KIRC, TCGA combined with GTEx also indicated that ZC3H13 was significantly lower in tumor tissues (**Supplementary Figure 1B**). In addition, **Supplementary Figure 1C** shows the expression level of ZC3H13 in various normal tissues in the GTEx database. We found that ZC3H13 had the lowest expression level in blood, which indicated that as a target for drug therapy, ZC3H13 might have low blood system toxicity and side effects. Finally, we also explored the expression of ZC3H13 in each tumor cell line in the CCLE database as shown in **Supplementary Figure 1D**.

### Prognostic Significance and Immunological Role of ZC3H13 in Pan-Cancers

The differential expression patterns of ZC3H13 in pan-cancers prompted us to explore its prognostic value. Therefore, we performed survival analyses in pan-cancers in terms of overall survival (OS), progression-free survival (PFS) and cancer-specific survival (CSS) by using the Cox regression model, Kaplan-Meier analysis and log-rank test. For OS, high expression of ZC3H13 was associated with favorable prognosis in KIRC and poor prognosis in CESC (**Supplementary Figure 2**). For PFS, high expression of ZC3H13 was also associated with favorable prognosis in KIRC, kidney renal papillary cell carcinoma (KIRP) and prostate adenocarcinoma (PRAD) and poor prognosis in CESC (**Supplementary Figure 3**). Similarly, for CSS, high expression of ZC3H13 was still associated with favorable prognosis in KIRC, KIRP, and thymoma (THYM) and poor prognosis in CESC (**Supplementary Figure 4**). There is clear heterogeneity in the prognostic value of ZC3H13 in different tumors. In CESC, high expression of ZC3H13 was associated with poor prognosis regardless of OS, PFS or CSS, which suggested that ZC3H13 might be a carcinogenic factor in CESC. It is worth noting that the expression analysis from TCGA-CESC data indicated that ZC3H13 was significantly expressed at lower levels in CESC tumor tissues (**Supplementary Figure 1A**). This result suggested that ZC3H13 was more likely to be a tumor suppressor in CESC. More importantly, there was no significant difference in the expression of ZC3H13 between cancer and adjacent tissues when combining the TCGA-CESC and GTEx databases (**Supplementary Figure 1B**). However, high expression of ZC3H13 was associated with favorable prognosis regardless of OS, PFS or CSS. In line with this result, ZC3H13 was also significantly expressed at low levels in KIRC tumor tissues. Therefore, we choose KIRC for further research.

To explore whether ZC3H13 could be a predictor for immunotherapy, we analyzed the relationship between ZC3H13 and multiple immune checkpoint inhibitors (ICIs) and TIICs. As shown in **Supplementary Figure 5A**, ZC3H13 was significantly related to the expression level of immune checkpoint molecules in most tumors. Additionally, ZC3H13 was significantly related to TIICs in most tumor microenvironments (**Supplementary Figure 5B**). TMB and MSI are the most accurate markers for predicting the efficacy of ICB so far. The higher the TMB and MSI scores are, the more sensitive the tumor is to the efficacy of ICB. Here, we found that ZC3H13 was significantly related to the TMB and MSI of many types of tumors. For example, ZC3H13 was negatively correlated with the MSI scores of BRCA, THCA, PRAD, HNSC, and DLBC, but it was positively correlated with the MSI scores of READ, OV and LUSC (**Supplementary Figure 5C**). ZC3H13 was significantly negatively correlated with TMB in KIRC, BRCA, THCA, STAD, PRAD, LUSC, and LIHC. However, ZC3H13 was significantly positively correlated with TMB in SKCM (**Supplementary Figure 5D**). All of these results suggested that ZC3H13 might have the potential to be a predictor of ICB efficacy.

### The Relationship Between ZC3H13 and Clinicopathological and Prognostic Characteristics in KIRC

**Figure 1:**
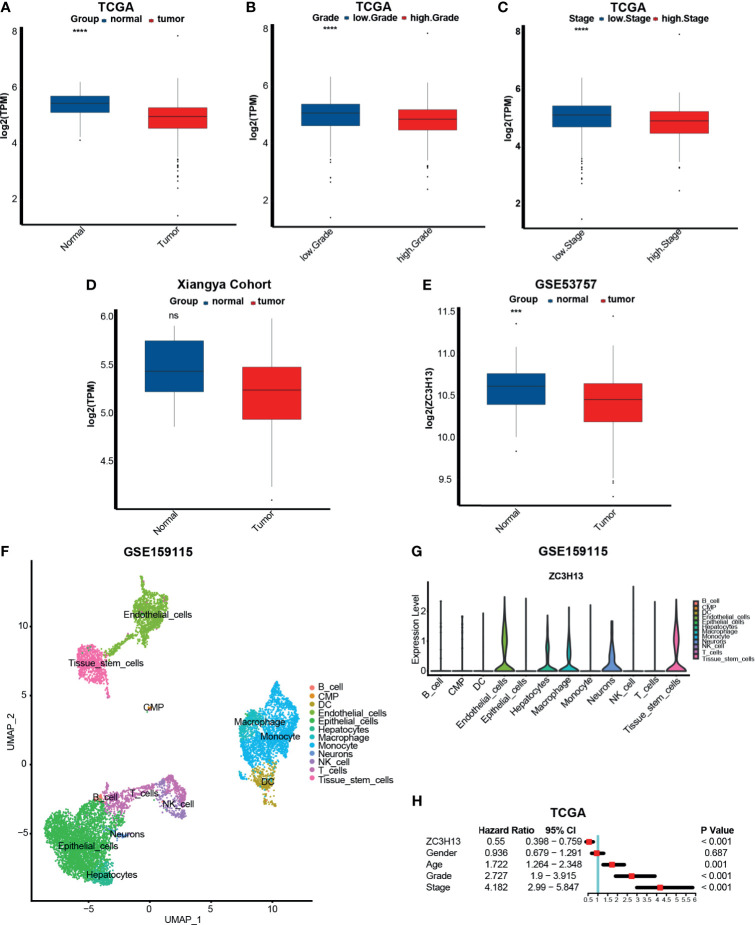
The relationship between ZC3H13 and clinicopathological and prognostic characteristics in KIRC. **(A)** The histogram of log2(TPM) of ZC3H13 between normal and cancer tissues based on TCGA database. Normal tissue, blue; Cancer tissue, red. (T test, ****P < 0.0001). **(B)** The histogram of log2(TPM) of ZC3H13 between low and high grade based on TCGA database. Low grade, blue; High grade, red. (T test, ****P < 0.0001).. **(C)** The histogram of log2(TPM) of ZC3H13 between low and high stages based on TCGA database. Low stage, blue; High stage, red. (T test, ****P < 0.0001). **(D)** The histogram of log2(TPM) of ZC3H13 between normal and cancer tissues in Xiangya cohort. Normal tissue, blue; Cancer tissue, red. (T test, ns, not statistically significant). **(E)** The histogram of log2(ZC3H13) between normal and cancer tissues based on GSE53757. Normal tissue, blue; Cancer tissue, red. (T test, ****P < 0.0001). **(F)** Single-cell atlas of KIRC adjacent normal tissues. UMAP plot of 6046 cells obtained from GSE159115, which was visualized and annotated using “Seurat” and “Single” R package respectively. CMP, common myeloid progenitor; DC, dendritic cell. **(G)** Violin plot of ZC3H13 expression pattern between different cell types in KIRC adjacent normal tissues. **(H)** Forest figure of single factor Cox analysis on sex, age, ZC3H13 expression, grade and stage. Calculated using Cox proportional hazard model and visualized using “forestplot” R package.

Based on the previous results, we further analyzed the correlation between ZC3H13 and some important clinicopathological characteristics here. In line with the previous results, we found that the expression of ZC3H13 in tumor tissues, higher grade and higher stage was significantly lower (**Figures 1A–C**). In our own RNA-seq cohort, though without significant difference, there was a trend that the expression was higher in the normal tissues (**Figure 1D**). And this no significant difference may be caused by the small sample size. To eliminate the influence of sample size, we chose a large GEO database (GSE53757), which contains 72 KIRC tumor tissues and matched adjacent normal tissues, and successfully validated this result (**Figure 1E**). As we found that ZC3H13 was significantly higher expressed in the normal tissues, we further explored which cell types ZC3H13 expressed in adjacent normal tissues using scRNA-seq. To our surprise, ZC3H13 was almost not expressed in T and NK cells and expressed abundantly in endothelial cells, macrophage, neurons and tissue stem cells (**Figures 1F, G**). The expression of ZC3H13 of these cells might inhibit T and NK cells from infiltrating into the tumor microenvironment as ZC3H13 was negatively correlated with the infiltration of TIICs in KIRC (**Supplementary Figure 5B**). Finally, we conducted a single factor Cox analysis on sex, age, ZC3H13 expression, grade and stage. The results suggested that older age, higher grade and stage, and lower expression of ZC3H13 were all unfavorable prognostic factors (**Figure 1H**).

### Identifying DEGs Between the High and Low ZC3H13 Groups and Functional Analyses of DEGs

**Figure 2:**
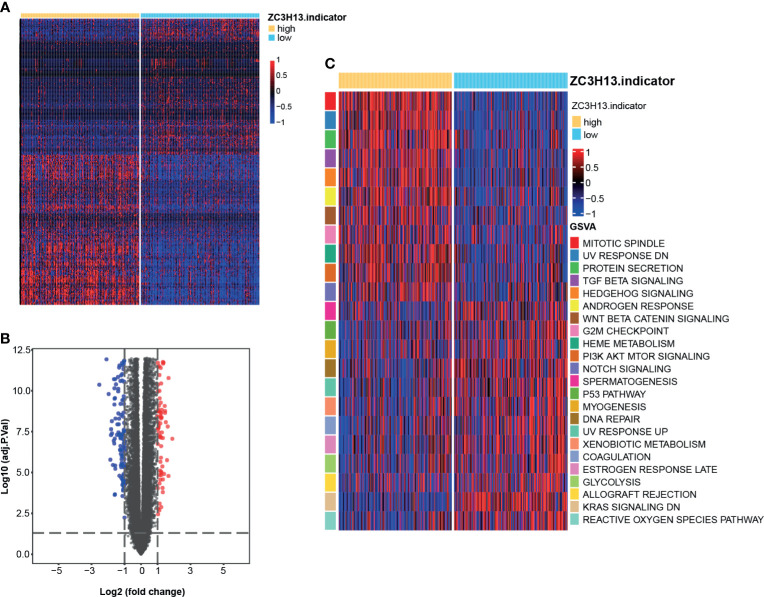
DEGs between the high and low ZC3H13 groups and functional analyses of DEGs. **(A)** Heatmap drawn based on the 271 DEGs between the high and low ZC3H13 groups. Lowly expressed DEGs, blue; Highly expressed DEGs, red. (“limma” R package, adjusted P value < 0.05 and |logFC| > 1 were set as the significance criteria for significant DEGs). **(B)** Volcano plot drawn based on the DEGs between the high and low ZC3H13 groups. Log2(FC) < -1, blue; Log2(FC) > 1, red; (“limma” R package, adjusted P value < 0.05 and |logFC| > 1 were set as the significance criteria for significant DEGs). **(C)** Heatmap drawn based on the GSVA analysis of biological pathways between the high and low ZC3H13 groups. Inhibition pathways, blue; Activation pathways, red.

A heatmap and volcano plot (**Figures 2A, B**) were used to display the screened DEGs. Eventually, we identified 271 significant DEGs (**Supplementary Table 7**). The results of GO analysis suggested that these DEGs were enriched in several biological processes, including organic anion transport, apical part of cell, receptor ligand activity, apical plasma membrane, collagen-containing extracellular matrix, and anion transmembrane transporter activity (**Supplementary Figures 6A–C** and **Supplementary Table 8**). The results of KEGG analysis indicated that these DEGs were enriched in pathways such as neuroactive ligand-receptor interaction and cholesterol metabolism (**Supplementary Figure 6D** and **Supplementary Table 9**). Additionally, the enrichment scores of several hallmark signatures were significantly different between the high and low ZC3H13 groups. Mitotic spindle, UV response down, protein secretion, TGF-β signaling, Hedgehog signaling, androgen response, Wnt-β-Catenin signaling, G2M checkpoint, heme metabolism, PI3K-AKT-MTOR signaling and Notch signaling were enriched in the high ZC3H13 group. In contrast, spermatogenesis, p53 pathway, myogenesis, DNA repair, UV response up, xenobiotic metabolism, coagulation, estrogen response late, glycolysis, allograft rejection, Kras signaling down, and reactive oxygen species pathway were enriched in the low ZC3H13 group (**Figure 2C** and **Supplementary Table 10**).

### ZC3H13 Shaped a Noninflamed Phenotype and Predicted a Lower Response to ICB in KIRC

**Figure 3:**
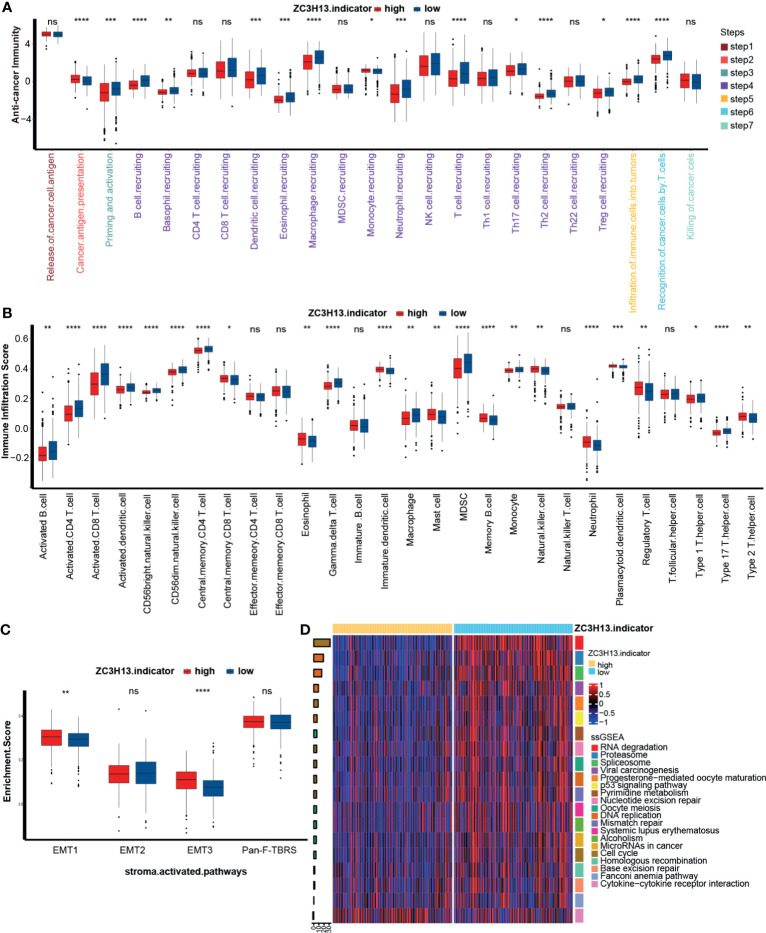
Different immunological characteristics between the high and low ZC3H13 groups **(A)** Activation of cancer immunity cycles between the high and low ZC3H13 groups; Low ZC3H13 group, blue; High ZC3H13 group, red. MDSC, myeloid-derived suppressor cell; NK, natural killer cell; Th, helper T cell; Treg, regulatory T cell. (T test, *P < 0.05; **P < 0.01; ***P < 0.001; ****P < 0.0001; ns, not statistically significant). **(B)** The scores of immune cell infiltration in the TME between the high and low ZC3H13 groups; Low ZC3H13 group, blue; High ZC3H13 group, red; MDSC, myeloid-derived suppressor cell. (T test, *P < 0.05; **P < 0.01; ***P < 0.001; ****P < 0.0001; ns, not statistically significant). **(C)** Activation of stroma-activated pathways between the high and low ZC3H13 groups; Low ZC3H13 group, blue; High ZC3H13 group, red; EMT, epithelial–mesenchymal transition; Pan-F-TBRS, panfibroblast TGF-b response signature. (GSVA analysis and T test, *P < 0.05; **P < 0.01; ***P < 0.001; ****P < 0.0001; ns, not statistically significant). **(D)** Heatmap based on different immunotherapy predicted pathways between the high and low ZC3H13 groups. The bar plots on the left represent log10 p-values; positive values, activation; negative values, inhibition; the bar plots on the right represent different pathways.

The previous results indicated that ZC3H13 is closely related to the immune characteristics of a variety of tumors. We further compared the different activities of the immune response between the ZC3H13 high and low groups. As shown in **Figure 3A**, the activities of the majority of immune cycles were downregulated in the high ZC3H13 group, including the activities of priming, activation and trafficking of immune cells to tumors (macrophage recruitment, NK cell recruitment, DC recruitment, and TH17 recruitment). In addition, the activities of infiltration of immune cells into tumors and recognition of cancer cells by T cells were also significantly lower in the high ZC3H13 group. The recruiting ability of CD8 T cells was also lower in the high ZC3H13 group, although there was no significant difference. To further verify these results, we applied the ssGSEA algorithm to calculate the infiltration levels of various immune cells in the TME. In line with previous results, the infiltration level of anticancer immune cells, including activated CD4 T cells, activated CD8 T cells, activated dendritic cells, CD56 bright natural killer cells, central memory CD4 T cells, macrophages, type 1 T helper cells, and type 17 T helper cells, was significantly lower in the high ZC3H13 group. Additionally, the infiltration level of protumor immune cells, such as regulatory T cells, plasmacytoid dendritic cells, neutrophils, and type 2 T helper cells, was significantly higher in the high ZC3H13 group. These results suggested that high ZC3H13 promoted the formation of a noninflamed phenotype (**Figure 3B**). It is well known that significant activation of the stromal pathway can inhibit tumor immunity and promote the formation of a noninflamed phenotype. We further found that the enrichment score of stromal pathways, including EMT1 and EMT3, was significantly higher in the high ZC3H13 group. Although there was no significant difference, the enrichment score of Pan-F-TBRS was also higher in the high ZC3H13 group (**Figure 3C**).

**Figure 4:**
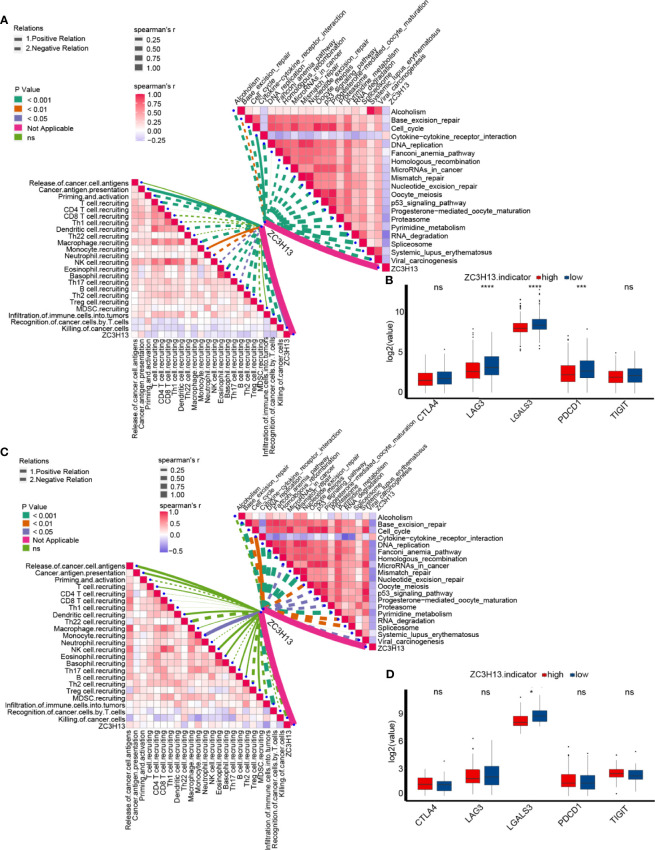
The linear relationship between the expression of ZC3H13 and the enrichment scores of immune cycles and ICB efficacy prediction pathways. **(A)** Spearman correlation of ZC3H13 expression with cancer immunity and immune related pathways, presented on the left and right respectively. The different types of lines represent positive or negative correlations; the thickness of the lines and the color of the bar plots represent the strength of correlation; and the different colors of the lines represent p-values. MDSC, myeloid-derived suppressor cell; NK, natural killer cell; Th, helper T cell; Treg, regulatory T cell. **(B)** The histogram of log2(TPM) values of immune checkpoint genes between different ZC3H13 groups. Low ZC3H13 group, blue; High ZC3H13 group, red. (T test, ***P < 0.001; ****P < 0.0001; ns, not statistically significant). **(C)** Spearman correlation of ZC3H13 expression with cancer immunity and immune related pathways in our own RNA-seq cohort, presented on the left and right respectively. The different types of lines represent positive or negative correlations; the thickness of the lines and the color of the bar plots represent the strength of correlation; and the different colors of the lines represent p-values. MDSC, myeloid-derived suppressor cell; NK, natural killer cell; Th, helper T cell; Treg, regulatory T cell. **(D)** The histogram of log2(TPM) values of immune checkpoint genes between different ZC3H13 groups in our own RNA-seq cohort. Low ZC3H13 group, blue; High ZC3H13 group, red. (T test, *P < 0.05; ns, not statistically significant).

An inflamed tumor microenvironment (TME) in conjunction with pre-existing anticancer immunity is necessary for ICB (23–26). Therefore, we further analyzed the difference in enrichment scores of ICB efficacy prediction pathways between the high and low ZC3H13 groups. As expected, the enrichment scores of pathways that were positively related to the response to ICB were significantly lower in the high ZC3H13 group, such as nucleotide excision repair, oocyte meiosis, DNA replication, mismatch repair, systemic lupus erythematosus, alcohol, microRNAs in cancer, and the cell cycle (**Figure 3D** and **Supplementary Figure 6F**). Additionally, the enrichment scores of the cytokine-cytokine receptor pathway, which was negatively related to the response to ICB, were significantly higher in the high ZC3H13 group (**Figure 3D** and **Supplementary Figure 6F**). Furthermore, we analyzed the linear relationship between the expression of ZC3H13 and the enrichment scores of these immune cycles and ICB efficacy prediction pathways. ZC3H13 was still significantly negatively correlated with the enrichment scores of the antitumor immune signatures (**Figure 4A** left, ** Supplementary Figure 7** and **Supplementary Table 11**) and ICB efficacy prediction pathways (**Figure 4A** right, **Supplementary Figure 8** and **Supplementary Table 12**). The expression of several critical immune checkpoints, including CTLA-4, PD-1, LAG-3, LAALS3 and TIGIT, was significantly higher in the low ZC3H13 group (**Figure 4B**). Then, we validated these results in our RNA-seq cohort. ZC3H13 was significantly negatively correlated with the ICB efficacy prediction pathways (**Figure 4C** right, ** Supplementary Figure 9** and **Supplementary Table 13**) and most of the enrichment scores of the antitumor immune signatures (**Figure 4C** left, ** Supplementary Figure 10** and **Supplementary Table 14**). Finally, we also validated that LAALS3 was significantly higher in the low ZC3H13 group (**Figure 4D**).

In summary, ZC3H13 may be a novel biomarker to predict the immune phenotypes and clinical response of ICB in KIRC.

### The Relationship Between ZC3H13 and Tumor Mutation Spectrum, TMB, and MSI in KIRC

**Figure 5:**
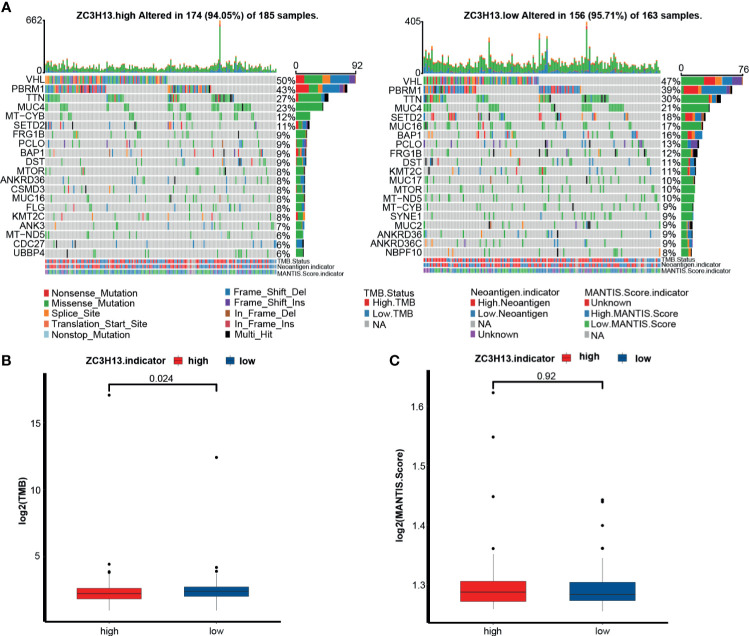
The relationship between ZC3H13 and the tumor mutation spectrum, TMB and MSI in KIRC. **(A)** Mutation spectrum of the high (left) and low (right) ZC3H13 groups in KIRC. Different colors represented different mutation types annotated at the bottom; The barplot on the top represented mutation burden. The numbers on the right represented mutation frequency. TMB, tumor mutation burden. MANTIS, microsatellite analysis for normal-tumor instability. **(B)** The histogram of log2(value) of TMB between the different ZC3H13 groups. Low ZC3H13 group, blue; High ZC3H13 group, red. (T test). **(C)** The histogram of log2(value) of MANTIS score between the different ZC3H13 groups. Low ZC3H13 group, blue; High ZC3H13 group, red. (T test).

Here, we compared the distribution differences of the top 20 somatic mutations between ZC3H13 groups. Notably, VHL, PBRM1 and TNN were the most frequent mutations in KIRC (**Figure 5A**). The overall mutational profiles between the ZC3H13 groups were comparable (94.5% *vs* 95.7%). Despite this, TMB in the low ZC3H13 group was significantly higher than that in the high ZC3H13 group (**Figure 5B**). However, there was no significant difference in the MSI scores between the two groups (**Figure 5C**).

### Role of ZC3H13 in Predicting the Sensitivity of Targeted Therapy for KIRC

**Figure 6:**
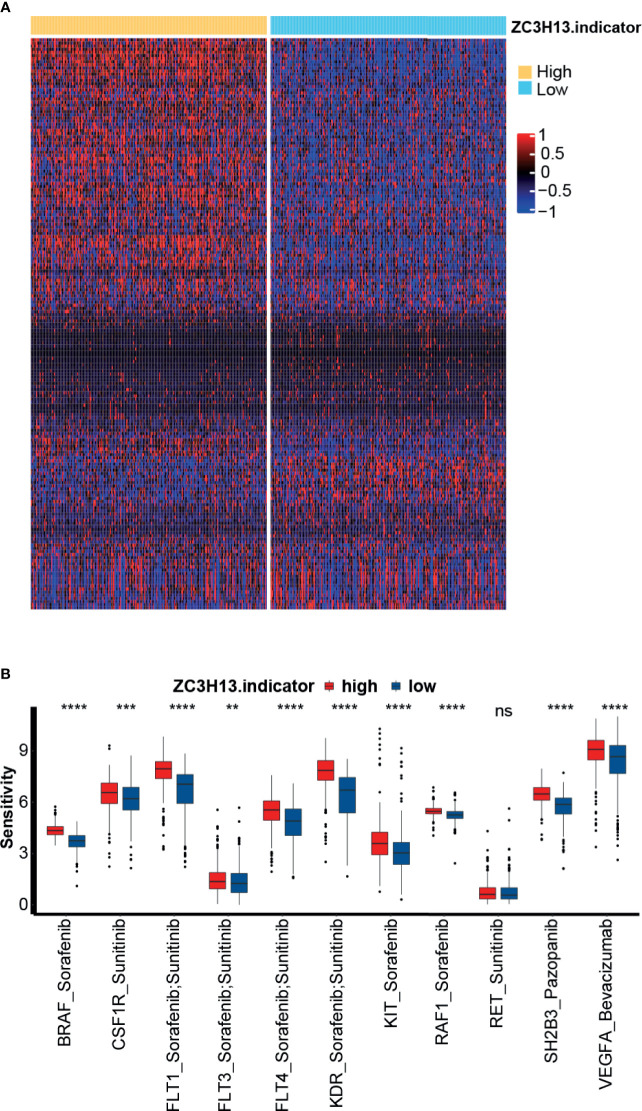
The relationship between ZC3H13 and the sensitivity to targeted therapy of KIRC. **(A)** Heatmap drawn based on the different sensitivities to the 183 drugs selected from the DrugBank database. **(B)** The histogram of sensitivities to the selected targeted therapy between the different ZC3H13 groups. Low ZC3H13 group, blue; High ZC3H13 group, red. (T test, **P < 0.01; ***P < 0.001; ****P < 0.0001; ns, not statistically significant).

Targeted therapy is the most important treatment option for KIRC. We selected 183 drugs for the treatment of solid tumors and the corresponding target genes from the DrugBank database. Then, we compared the sensitivity of these antitumor drugs between the high and low ZC3H13 groups. As shown in **Figure 6A** and **Supplementary Table 15**, the sensitivity of most drugs was significantly different between the two groups. Furthermore, we focused on several targeted therapies and genes that were most commonly used in advanced KIRC patients: sorafenib with its targeted genes, including BRAF, FLT1, FLT3, FLT4, KDR, KIT, and RAF1; sunitinib with its targeted genes, including CSF1R, FLT1, FLT3, FLT4, KDR, and RET; pazopanib with its targeted gene SH2B3; and bevacizumab with its targeted gene VEGFA. We found that the sensitivity of these drugs was significantly higher in the high ZC3H13 group (**Figure 6B**). This finding indicated that targeted therapy could be a treatment option for the high ZC3H13 group, though this group was less sensitive to ICB therapy.

## Discussion

This study comprehensively analyzed the different expression profiles, prognostic values and immunoregulatory effects of ZC3H13 in pan-cancers. We found that ZC3H13 was closely related to the occurrence of a variety of tumors, especially KIRC. Then, we focused the analyses of ZC3H13 on KIRC. ZC3H13 might be a tumor suppressor gene in KIRC. Interestingly, the high expression of KIRC represented a noninflamed phenotype and this result could be roughly validated in our own RNA-seq cohort. Patients with high ZC3H13 expression were less sensitive to ICB but were more sensitive to targeted therapy. These results suggested that ZC3H13 was a potential predictive marker for ICB and targeted therapy in KIRC.

Given the substantial economic burden and toxic side effects, it is vital to find more reliable and simpler ICB efficacy prediction markers. To date, some ICB efficacy prediction markers have been identified, including PD-L1, TMB, MSI and some other efficacy prediction models, such as the TIDE model (27). However, it is worth noting that all these predictive markers have encountered many obstacles in clinical practice. The most serious obstacle is that the prediction accuracy is not sufficient. For example, as a marker for predicting the efficacy of ICB, the accuracy of PD-L1 can be affected by many other factors, such as immunohistochemical test methods, detection antibodies, and the choice of positive threshold (28, 29). TMB and MSI have relatively higher accuracy in predicting the efficacy of ICB than PD-L1. However, the clinical detection of these two markers relies on expensive and complex molecular methods. The tumor microenvironment (TME) plays an important role in tumor immunotherapy. An inflamed TME in conjunction with pre-existing anticancer immunity is necessary for ICB (23–26). Therefore, finding a biomarker that can fully predict the immune phenotype opens a new road for predicting the efficacy of ICB. In this study, we found that ZC3H13 could predict the immune phenotype from multiple angles.

First, we indicated that ZC3H13 was significantly correlated with the activity of the antitumor immune response steps in the TME of KIRC (19). The activities of the major cycles were downregulated in the high ZC3H13 group, including the activities of priming and activation, trafficking of immune cells to tumors (macrophage recruitment, NK cell recruitment, DC recruitment, and TH17 recruitment), infiltration of immune cells into tumors, and recognition of cancer cells by T cells. This indicated that ZC3H13 could inhibit the body’s immune monitoring of tumor cells from the origin and further promote the immune evasion of tumor cells. The types of immune cells in the tumor microenvironment are complex, and their infiltration varies greatly. In KIRC, high expression of ZC3H13 could significantly inhibit the infiltration of most tumor suppressor TIICs, including activated CD4 T cells, activated CD8 T cells, activated dendritic cells, CD56 bright natural killer cells, central memory CD4 T cells, type 1 T helper cells, and type 17 T helper cells. Additionally, the infiltration of cancer-promoting TIICs, including regulatory T cell, plasmacytoid dendritic cells, neutrophils, and type 2 T helper cells, was significantly increased in the high ZC3H13 group. In addition, the activation of stromal pathways could also affect antitumor immunity in the TME. We found that the stromal pathways (including EMT1 and EMT3) in the high ZC3H13 group were significantly activated. In summary, we have proven from multiple angles that high expression of ZC3H13 represents a noninflamed phenotype.

Since high expression of ZC3H13 can predict a noninflamed phenotype, patients with high ZC3H13 expression may not be sensitive to ICB treatment. Unfortunately, we lacked a database containing patients treated with ICB to directly analyze the relationship between ZC3H13 and ICB efficacy. Therefore, we analyzed the relationship between ZC3H13 and the predictive pathways that were closely related to the efficacy of ICB (22). As expected, the enrichment scores of pathways that were positively related to the response to ICB, such as nucleotide excision repair, oocyte meiosis, DNA replication, mismatch repair, systemic lupus erythematosus, alcohol, microRNAs in cancer, and the cell cycle, were significantly lower in the high ZC3H13 group. In contrast, the enrichment score of the cytokine-cytokine receptor interaction, which was negatively related to the response to ICB, was significantly higher in the high ZC3H13 group. At the same time, we found that ZC3H13 and several critical immune checkpoints, such as CTLA-4, PD-1, LAG-3, LAALS3, and TIGIT, were also significantly negatively correlated. Most importantly, we found that ZC3H13 was also significantly negatively correlated with TMB in KIRC. The above results indicated that high expression of ZC3H13 could not only predict a noninflamed phenotype but also indicate a lower sensitivity to ICB. Nevertheless, patients with high expression of ZC3H13 were more sensitive to targeted therapy.

There are some limitations in the study. First, this study was based on an analysis of public databases and our small sample size RNA-seq cohort. Therefore, the conclusions need further verification in larger cohort, especially the cohort receiving ICB treatment. Second, this study chose the median expression of ZC3H13 as the cutoff value. This cutoff value may not be suitable for use in further external datasets. Third, further mechanistic experiments are still needed to clarify the immunoregulatory effects of ZC3H13 on the tumor microenvironment of KIRC.

## Conclusion

This study demonstrated that ZC3H13 might shape a noninflamed phenotype in KIRC. Moreover, ZC3H13 could predict the prognosis and clinical response of ICB and the sensitivity to targeted therapies in KIRC.

## Data Availability Statement

The datasets presented in this study can be found in online repositories. The names of the repository/repositories and accession number(s) can be found in the article/**Supplementary Material**.

## Ethics Statement

The studies involving human participants were reviewed and approved by the Ethics Committee of the Xiangya Hospital of Central South University. The patients/participants provided their written informed consent to participate in this study.

## Author Contributions

(I) Conception and design: TG, HL, JH, and XZ. Administrative support: TG and XZ. Provision of study materials or patients: TG, JC, and JL. Collection and assembly of data: JL, BO, and HL. Data analysis and interpretation: HD, TG, HL, and JH. All authors contributed to the article and approved the submitted version.

## Funding

This work was supported by the Hunan Province Science and Technology Program (2018SK51714).

## Conflict of Interest

The authors declare that the research was conducted in the absence of any commercial or financial relationships that could be construed as a potential conflict of interest.

## Publisher’s Note

All claims expressed in this article are solely those of the authors and do not necessarily represent those of their affiliated organizations, or those of the publisher, the editors and the reviewers. Any product that may be evaluated in this article, or claim that may be made by its manufacturer, is not guaranteed or endorsed by the publisher.

## Supplementary Material

Supplementary Figure 1 Expression pattern of ZC3H13 in pan-cancers. **(A, B)** The expression pattern of ZC3H13 of pan-cancers in TCGA and TCGA combined with GTEx. The asterisks indicate a significant statistical p value calculated with the T test (*P < 0.05; **P < 0.01; ***P < 0.001). **(C)** The expression of ZC3H13 in normal tissues from the GTEx database. **(D)** The expression of ZC3H13 in cancer cell lines in CCLE.

Supplementary Figure 2 Prognostic analysis of ZC3H13 for overall survival in pan-cancers. **(A)** The prognostic analyses of ZC3H13 in pan-cancers using a univariate Cox regression model. A hazard ratio >1 indicated a risk factor, and a hazard ratio <1 represented a protective factor. **(B, C)** The prognostic analyses of ZC3H13 in pan-cancers using the Kaplan-Meier method and log-rank test. Only cancers in which ZC3H13 was a significant prognostic biomarker are shown.

Supplementary Figure 3 Prognostic analysis of ZC3H13 for progression-free survival in pan-cancers. **(A)** The prognostic analyses of ZC3H13 in pan-cancers using a univariate Cox regression model. A hazard ratio >1 indicats a risk factor, and a hazard ratio <1 represents a protective factor. **(B–E)** The prognostic analyses of ZC3H13 across cancers using the Kaplan-Meier method and log-rank test. Only cancers in which ZC3H13 was a significant prognostic biomarker are shown.

Supplementary Figure 4 Prognostic analysis of ZC3H13 for disease-specific survival in pan-cancers. **(A)** The prognostic analyses of ZC3H13 in pan-cancers using a univariate Cox regression model. A hazard ratio >1 indicates a risk factor, and a hazard ratio <1 represents a protective factor. **(B–E)** The prognostic analyses of ZC3H13 in pan-cancers using the Kaplan-Meier method and log-rank test. Only cancers in which ZC3H13 was a significant prognostic biomarker are shown.

Supplementary Figure 5 Correlations between ZC3H13 and immune checkpoints, tumor infiltrating immune cells, TMB, and MSI in pan-cancers. **(A)** Correlation between ZC3H13 and immune checkpoints in pan-cancers. **(B)** Correlation between ZC3H13 and MSI in pan-cancers. **(C)** Correlation between ZC3H13 and tumor infiltrating immune cells in pan-cancers. **(D)** Correlation between ZC3H13 and MSI in pan-cancers. The asterisks indicate a significant statistical p value calculated with Spearman correlation analysis (*P < 0.05; **P < 0.01; ***P < 0.001).

Supplementary Figure 6 Functional annotation for different expression genes between the high and low ZC3H13 groups. **(A)** Biological Processes (BP) **(B)** Cellular Components (CC); **(C)** Molecular Functions (MF); **(D)** Kyoto Encyclopedia of Genes and Genomes (KEGG). **(F)** The histogram of immunotherapy predicted pathways between the high and low ZC3H13 groups. Low ZC3H13 group, blue; High ZC3H13 group, red. (T test, *P < 0.05; **P < 0.01; ***P < 0.001; ****P < 0.0001; ns, not statistically significant).

Supplementary Figure 7 Spearman correlation of ZC3H13 expression with cancer immunity in TCGA cohort.

Supplementary Figure 8 Spearman correlation of ZC3H13 expression with immune related pathways in TCGA cohort.

Supplementary Figure 9 Spearman correlation of ZC3H13 expression with immune related pathways in our own cohort.

Supplementary Figure 10 Spearman correlation of ZC3H13 expression with cancer immunity in our own cohort.

## Abbreviations

TME, tumor immune microenvironment; ICB, immune checkpoint blockade; KIRC, kidney renal clear cell carcinoma; TMB, tumor mutation burden; m6A, RNA modification of N6-methyladenosine; ZC3H13, zinc finger CCCH domain-containing protein 13; TPM, transcripts per kilobase million; MSI, microsatellite instability; DEG, differentially expressed genes; GO, Gene Ontology; KEGG, Kyoto Encyclopedia of Genes and Genomes; TIIC, tumor-infiltrating immune cell; EMT, epithelial-mesenchymal transition; BLCA, bladder urothelial carcinoma; BRCA, breast invasive carcinoma; CESC, cervical squamous cell carcinoma and endocervical adenocarcinoma; CHOL, cholangiocarcinoma; ESCA, esophageal carcinoma; SKCM, skin cutaneous melanoma; KIRP, kidney renal papillary cell carcinoma; PRAD, prostate adenocarcinoma; THYM, thymoma; OS, overall survival; PFS, progression-free survival; CSS, cancer-specific survival; ICI, immune checkpoint inhibitor; MDSC, myeloid-derived suppressor cell; NK, natural killer cell; Th, helper T cell; Treg, regulatory T cell; EMT, epithelial–mesenchymal transition; Pan-F-TBRS, panfibroblast TGF-b response signature; MANTIS, microsatellite analysis for normal-tumor instability.
