# Supplementary figures and images for: N6-Methyladenosine Writer Gene ZC3H13 Predicts Immune Phenotype and Therapeutic Opportunities in Kidney Renal Clear Cell Carcinoma
Source: Front Oncol. 2021 Aug 23;11:718644. doi: 10.3389/fonc.2021.718644 (PMC8420859; doi:10.3389/fonc.2021.718644)

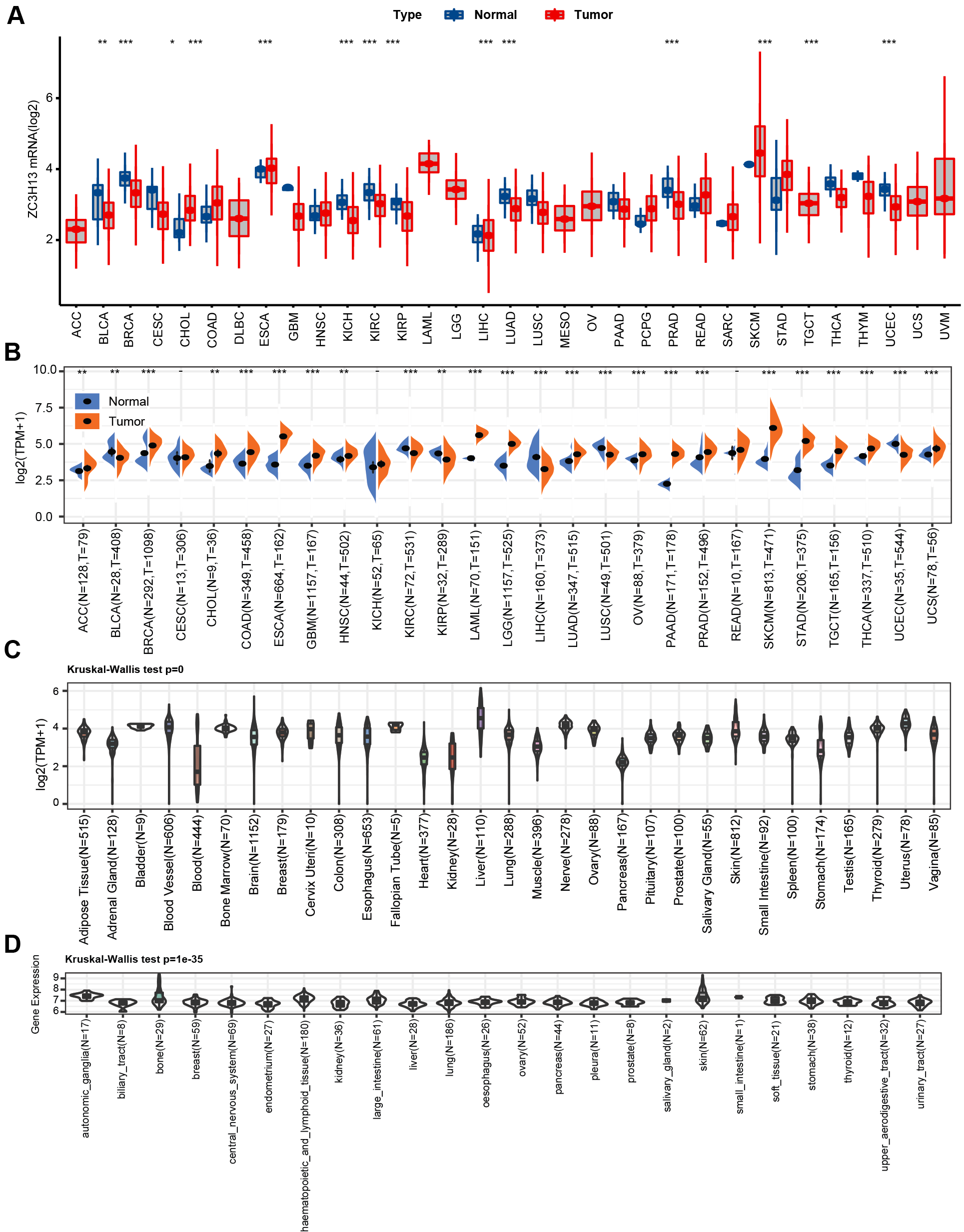

Supplement: Supplementary Figure 1 — Expression pattern of ZC3H13 in pan-cancers. (A, B) The expression pattern of ZC3H13 of pan-cancers in TCGA and TCGA combined with GTEx. The asterisks indicate a significant statistical p value calculated with the T test (*P < 0.05; **P < 0.01; ***P < 0.001). (C) The expression of ZC3H13 in normal tissues from the GTEx database. (D) The expression of ZC3H13 in cancer cell lines in CCLE. [file Image_1.tif]

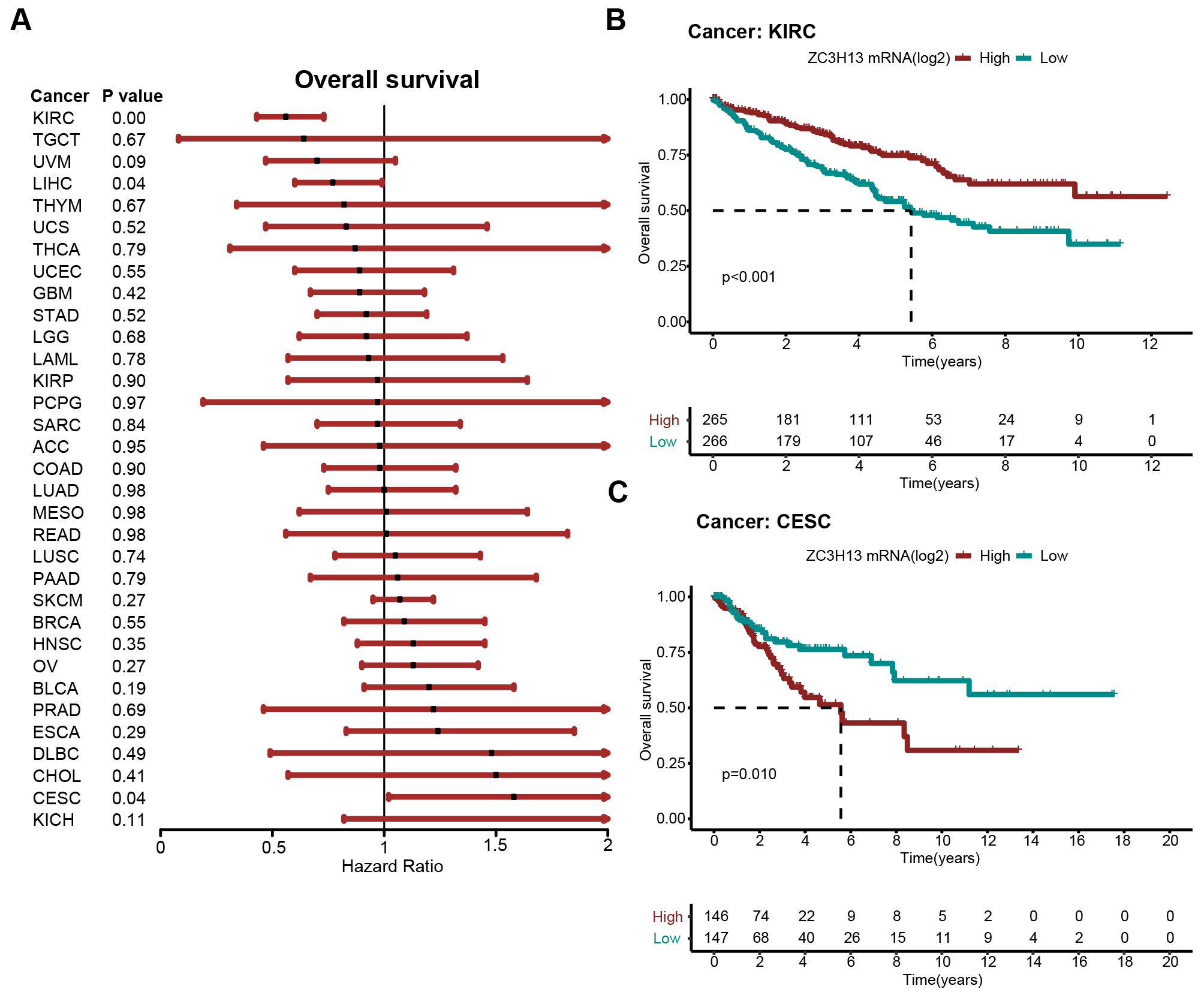

Supplement: Supplementary Figure 2 — Prognostic analysis of ZC3H13 for overall survival in pan-cancers. (A) The prognostic analyses of ZC3H13 in pan-cancers using a univariate Cox regression model. A hazard ratio >1 indicated a risk factor, and a hazard ratio <1 represented a protective factor. (B, C) The prognostic analyses of ZC3H13 in pan-cancers using the Kaplan-Meier method and log-rank test. Only cancers in which ZC3H13 was a significant prognostic biomarker are shown. [file Image_2.tif]

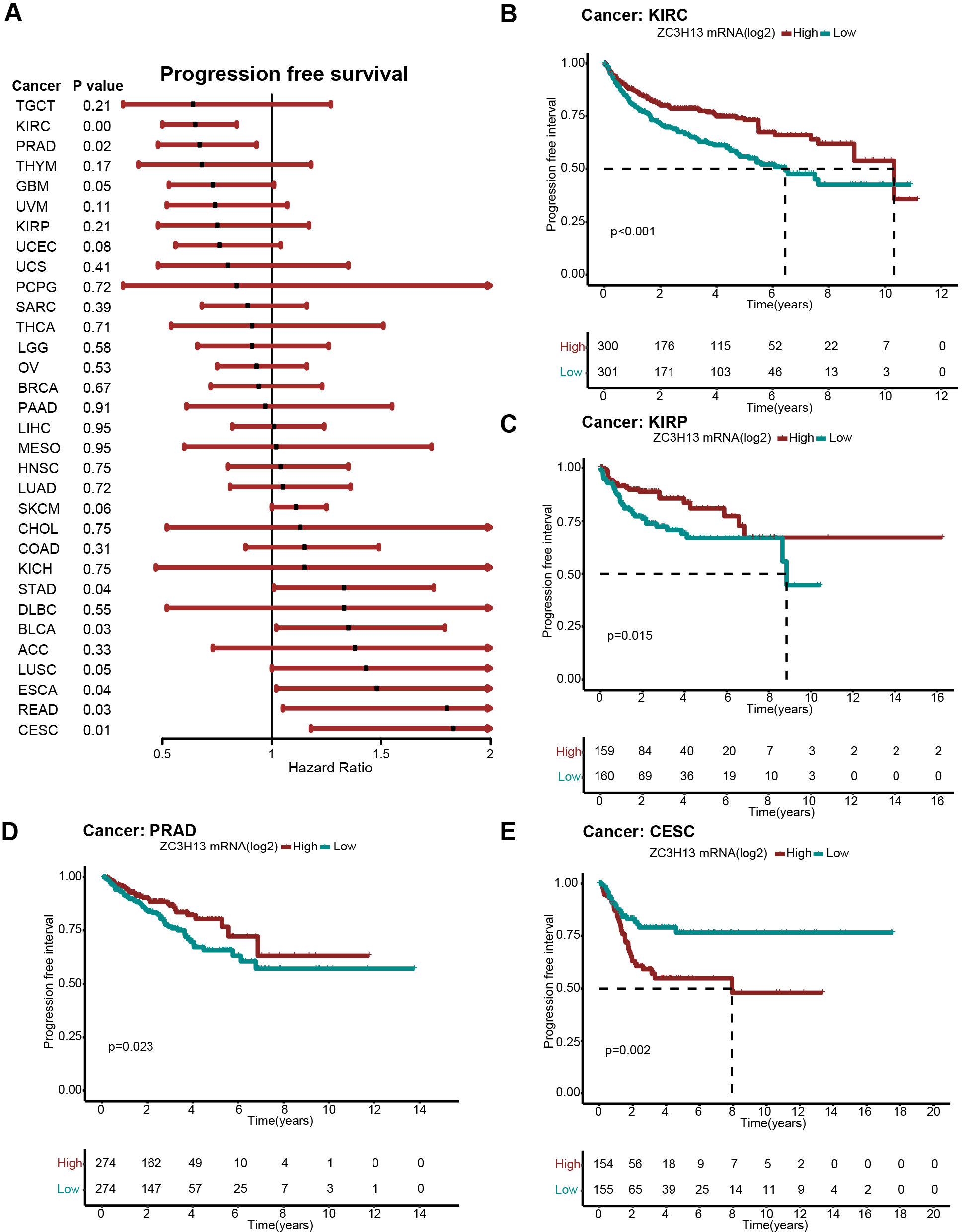

Supplement: Supplementary Figure 3 — Prognostic analysis of ZC3H13 for progression-free survival in pan-cancers. (A) The prognostic analyses of ZC3H13 in pan-cancers using a univariate Cox regression model. A hazard ratio >1 indicats a risk factor, and a hazard ratio <1 represents a protective factor. (B–E) The prognostic analyses of ZC3H13 across cancers using the Kaplan-Meier method and log-rank test. Only cancers in which ZC3H13 was a significant prognostic biomarker are shown. [file Image_3.tif]

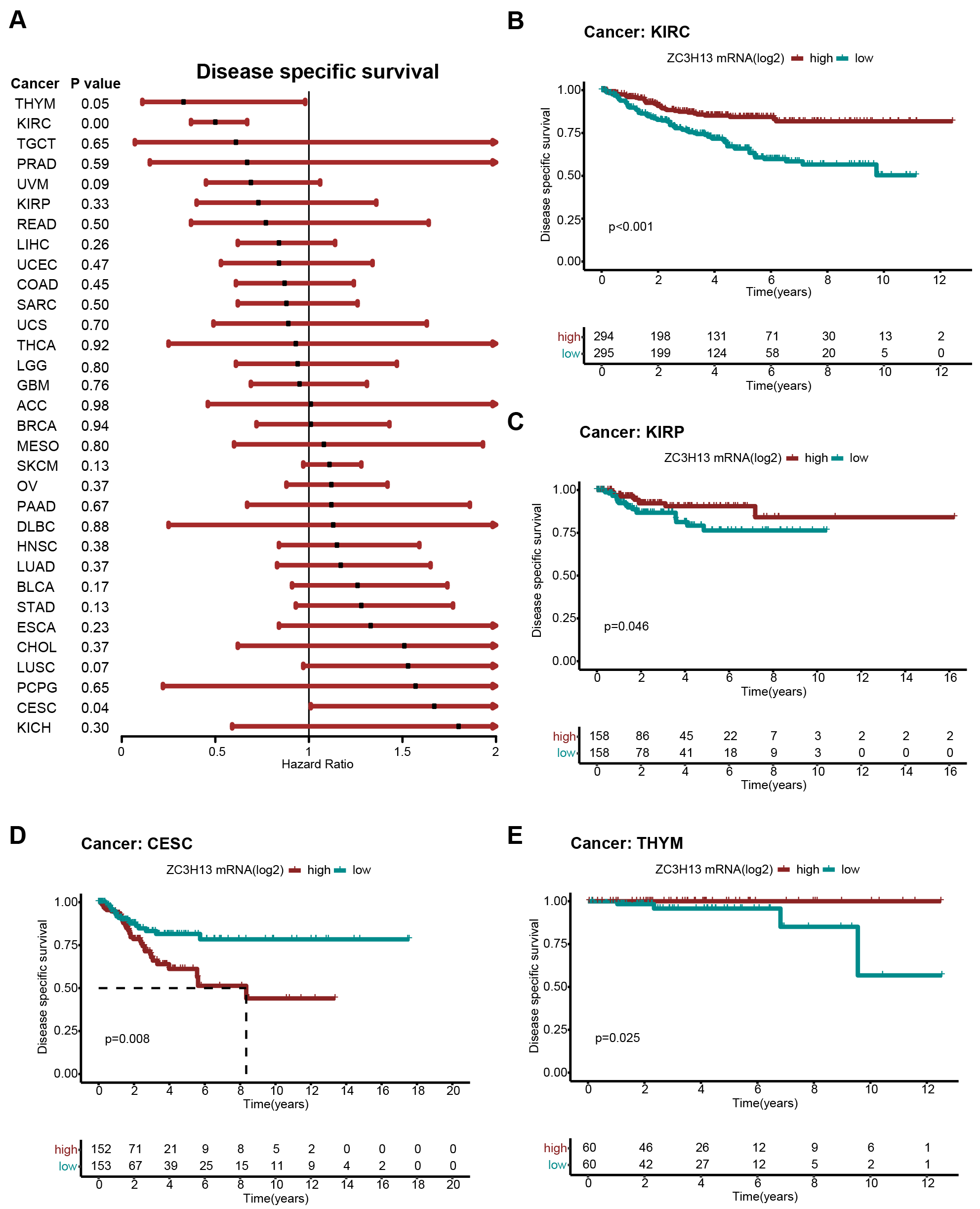

Supplement: Supplementary Figure 4 — Prognostic analysis of ZC3H13 for disease-specific survival in pan-cancers. (A) The prognostic analyses of ZC3H13 in pan-cancers using a univariate Cox regression model. A hazard ratio >1 indicates a risk factor, and a hazard ratio <1 represents a protective factor. (B–E) The prognostic analyses of ZC3H13 in pan-cancers using the Kaplan-Meier method and log-rank test. Only cancers in which ZC3H13 was a significant prognostic biomarker are shown. [file Image_4.tif]

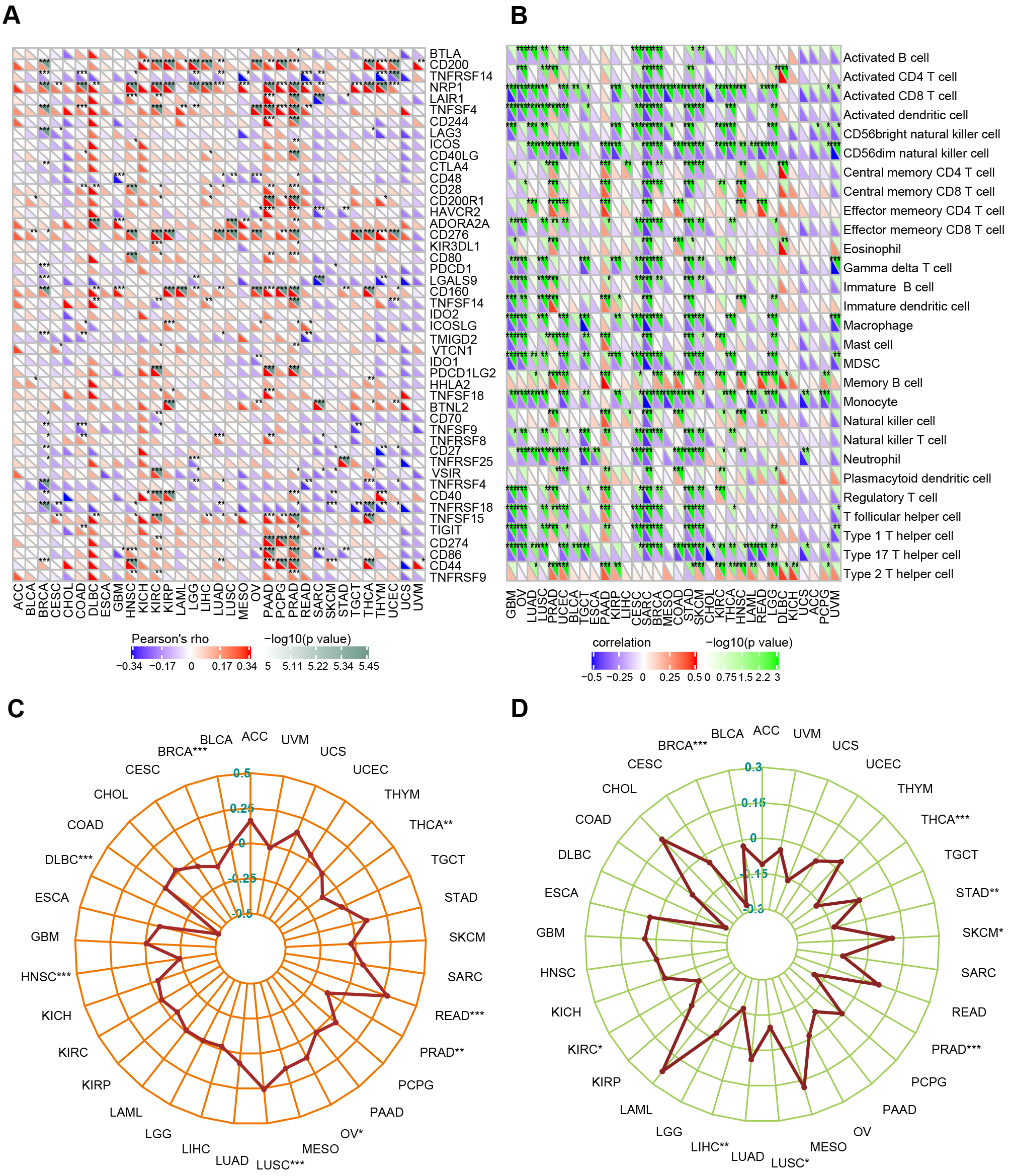

Supplement: Supplementary Figure 5 — Correlations between ZC3H13 and immune checkpoints, tumor infiltrating immune cells, TMB, and MSI in pan-cancers. (A) Correlation between ZC3H13 and immune checkpoints in pan-cancers. (B) Correlation between ZC3H13 and MSI in pan-cancers. (C) Correlation between ZC3H13 and tumor infiltrating immune cells in pan-cancers. (D) Correlation between ZC3H13 and MSI in pan-cancers. The asterisks indicate a significant statistical p value calculated with Spearman correlation analysis (*P < 0.05; **P < 0.01; ***P < 0.001). [file Image_5.tif]

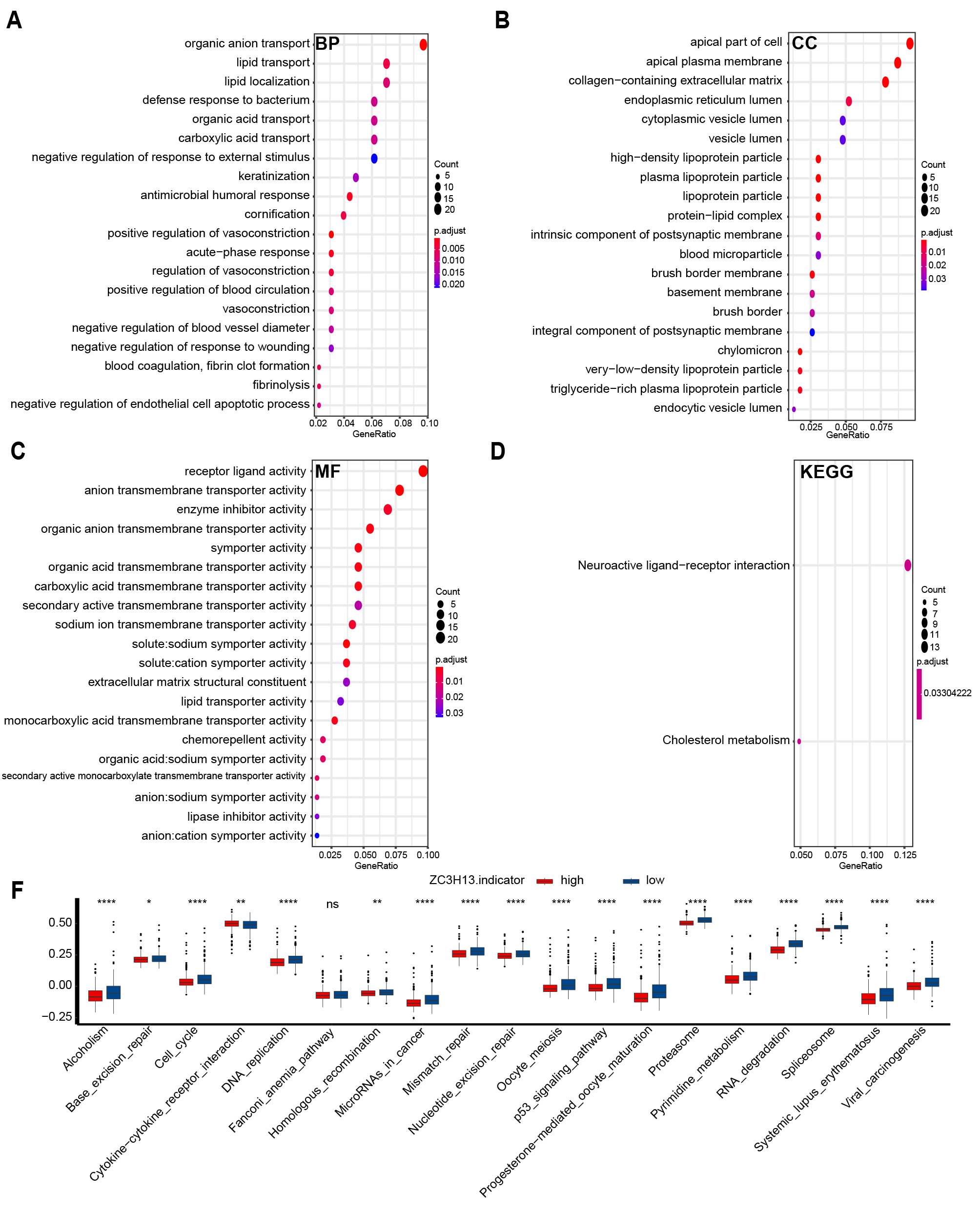

Supplement: Supplementary Figure 6 — Functional annotation for different expression genes between the high and low ZC3H13 groups. (A) Biological Processes (BP) (B) Cellular Components (CC); (C) Molecular Functions (MF); (D) Kyoto Encyclopedia of Genes and Genomes (KEGG). (F) The histogram of immunotherapy predicted pathways between the high and low ZC3H13 groups. Low ZC3H13 group, blue; High ZC3H13 group, red. (T test, *P < 0.05; **P < 0.01; ***P < 0.001; ****P < 0.0001; ns, not statistically significant). [file Image_6.tif]

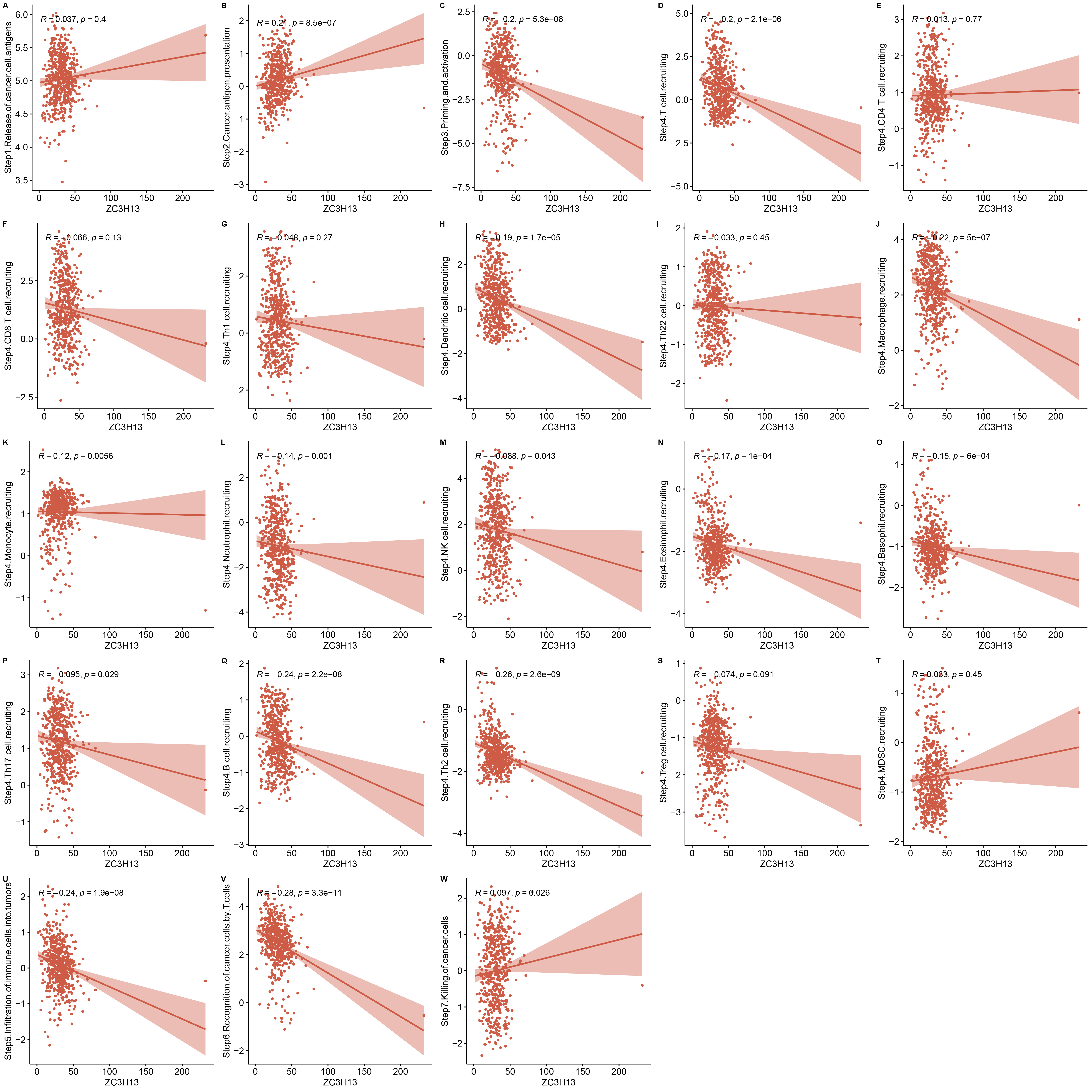

Supplement: Supplementary Figure 7 — Spearman correlation of ZC3H13 expression with cancer immunity in TCGA cohort. [file Image_7.tif]

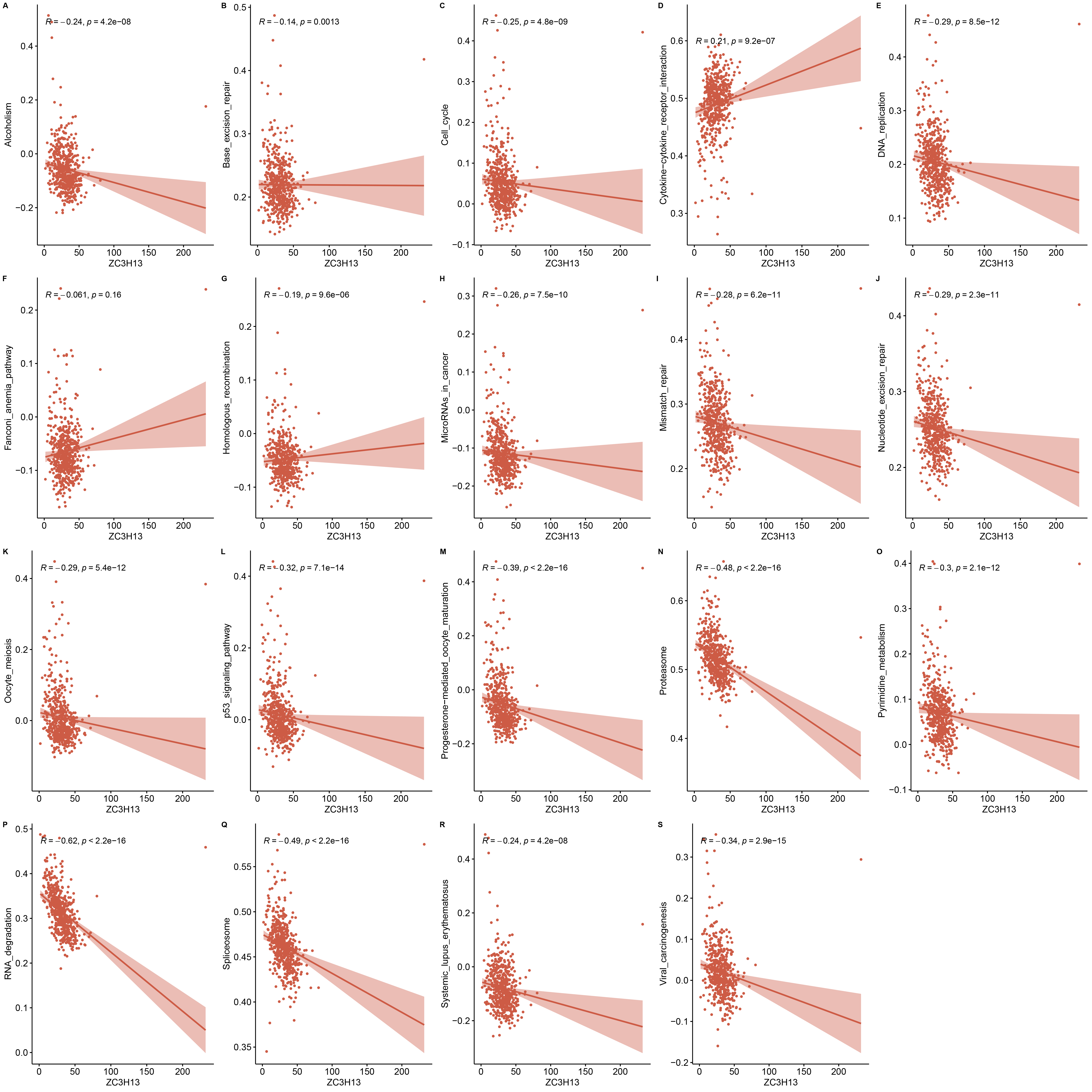

Supplement: Supplementary Figure 8 — Spearman correlation of ZC3H13 expression with immune related pathways in TCGA cohort. [file Image_8.tif]

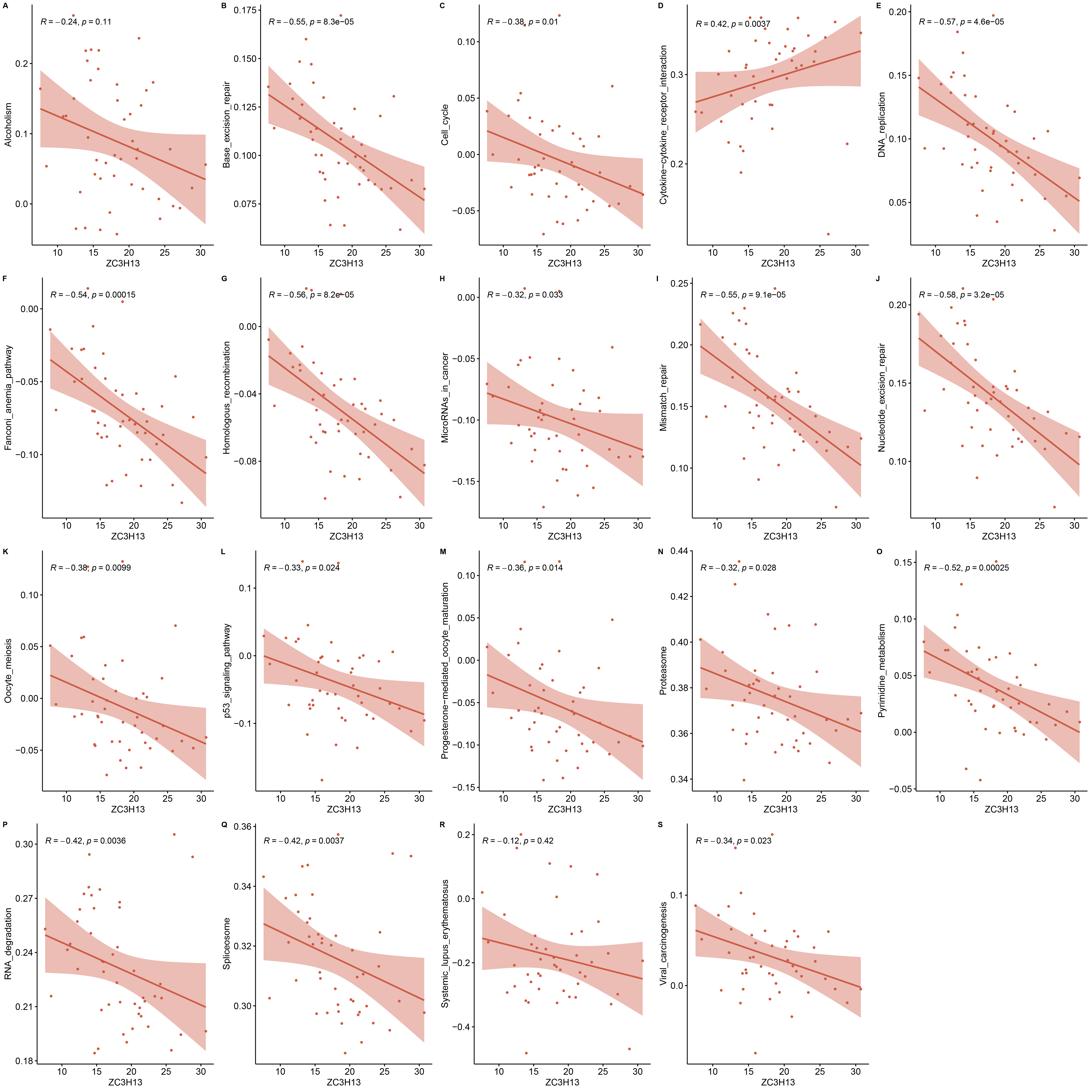

Supplement: Supplementary Figure 9 — Spearman correlation of ZC3H13 expression with immune related pathways in our own cohort. [file Image_9.tif]

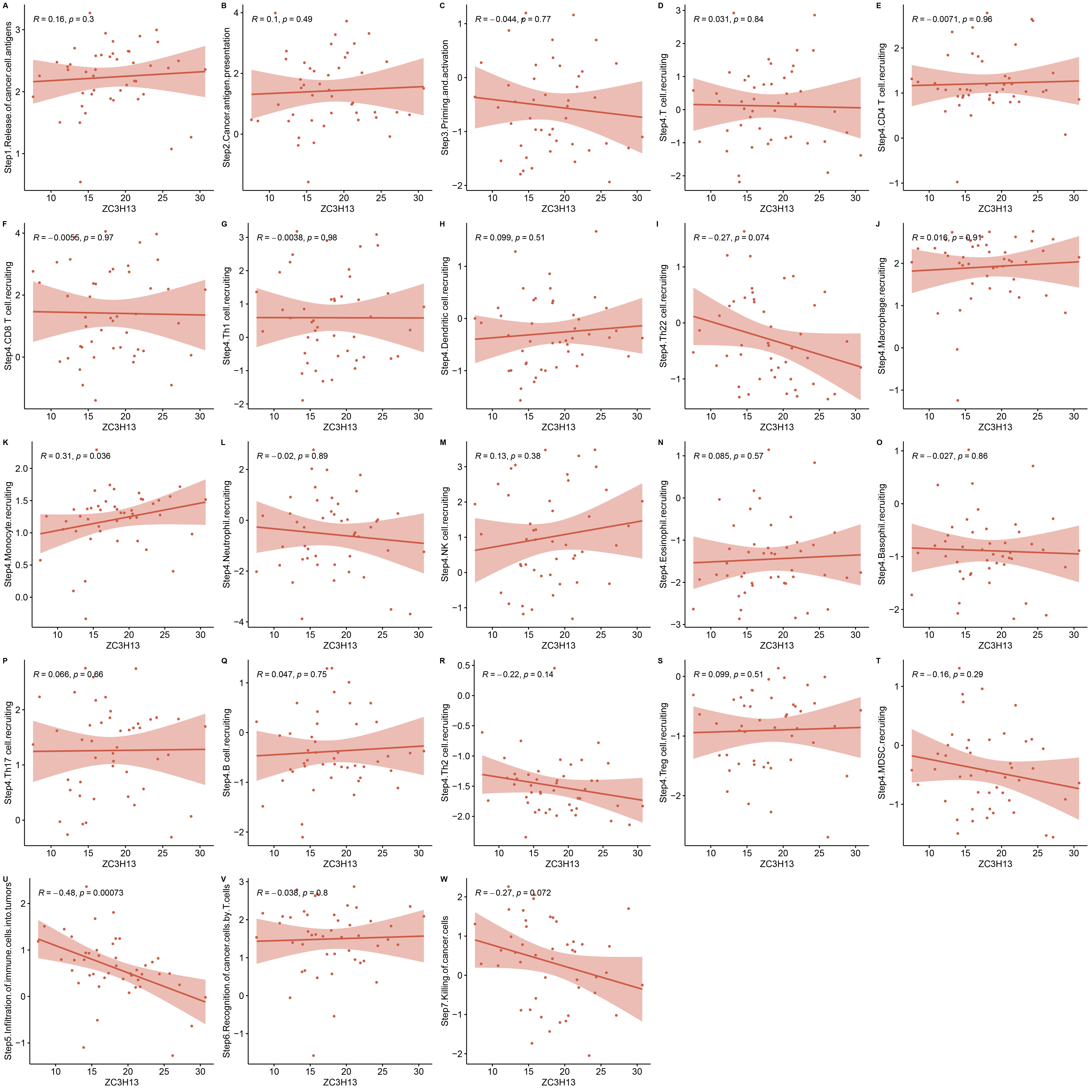

Supplement: Supplementary Figure 10 — Spearman correlation of ZC3H13 expression with cancer immunity in our own cohort. [file Image_10.tif]
